# One-carbon genetic variants and the role of *MTHFD1* 1958G>A in liver and colon cancer risk according to global DNA methylation

**DOI:** 10.1371/journal.pone.0185792

**Published:** 2017-10-02

**Authors:** Sara Moruzzi, Patrizia Guarini, Silvia Udali, Andrea Ruzzenente, Alfredo Guglielmi, Simone Conci, Patrizia Pattini, Nicola Martinelli, Oliviero Olivieri, Stephanie A. Tammen, Sang-Woon Choi, Simonetta Friso

**Affiliations:** 1 Department of Medicine, University of Verona School of Medicine, Verona, Italy; 2 Department of Surgery, Division of General and Hepatobiliary Surgery, University of Verona School of Medicine, Verona, Italy; 3 Friedman School of Nutrition Science and Policy, Tufts University, Boston, Massachusetts, United States of America; 4 Chaum Life Center, CHA University, Seoul, Korea; Universita degli Studi di Napoli Federico II, ITALY

## Abstract

Several polymorphic gene variants within one-carbon metabolism, an essential pathway for nucleotide synthesis and methylation reactions, are related to cancer risk. An aberrant DNA methylation is a common feature in cancer but whether the link between one-carbon metabolism variants and cancer occurs through an altered DNA methylation is yet unclear. Aims of the study were to evaluate the frequency of one-carbon metabolism gene variants in hepatocellular-carcinoma, cholangiocarcinoma and colon cancer, and their relationship to cancer risk together with global DNA methylation status. Genotyping for *BHMT* 716A>G, *DHFR* 19bp ins/del, *MTHFD1* 1958G>A, *MTHFR* 677C>T, *MTR* 2756A>G, *MTRR* 66A>G, *RFC1* 80G>A, *SHMT1* 1420C>T, *TCII* 776C>G and *TS* 2rpt-3rpt was performed in 102 cancer patients and 363 cancer-free subjects. Methylcytosine (mCyt) content was measured by LC/MS/MS in peripheral blood mononuclear cells (PBMCs) DNA. The *MTHFD1* 1958AA genotype was significantly less frequent among cancer patients as compared to controls (p = 0.007) and related to 63% reduction of overall cancer risk (p = 0.003) and 75% of colon cancer risk (p = 0.006). When considering PBMCs mCyt content, carriers of the *MTHFD1* 1958GG genotype showed a lower DNA methylation as compared to carriers of the A allele (p = 0.048). No differences were highlighted by evaluating a possible relationship between the other polymorphisms analyzed with cancer risk and DNA methylation.

The *MTHFD1* 1958AA genotype is linked to a significantly reduced cancer risk. The 1958GG genotype is associated to PBMCs DNA hypomethylation as compared to the A allele carriership that may exert a protective effect for cancer risk by preserving from DNA hypomethylation.

## Introduction

One-carbon metabolism is a complex pathway involved both in the nucleotide synthesis and biological methylation reactions [1, 2]. For the implication of this metabolism in cellular development, proliferation and differentiation, polymorphic variants of genes encoding for enzymes related to one-carbon metabolism have been largely studied for their relationship with cancer disease [3–6]. The molecular mechanisms underlying such association are, however, not clearly defined yet, while it is known that one-carbon metabolism modulates a major epigenetic mechanism as DNA methylation [7, 8] that is strongly linked to cancer [6]. DNA methylation is the major and the most studied among the epigenetic mechanisms in mammalian cells consisting in the covalent binding of a methyl group to the 5’ carbon position of cytosine, mostly occurring at CpG dinucleotide sequences with a function for gene expression regulation and maintainance of genomic stability [9]. DNA methylation is a heritable and reversible phenomenon with potential implications for the understanding of molecular mechanisms and disease prevention strategies in complex diseases such as cancer [6]. Both global and gene-specific DNA methylation show altered patterns in cancer disease and, in particular, a global DNA hypomethylation has been described as an almost universal finding in cancer cells [10]. There is evidence that global DNA hypomethylation in peripheral blood mononuclear cells (PBMCs) may be associated to an increased cancer risk and may be useful as a biomarker for cancer susceptibility [11]. Significantly reduced methylcytosine (mCyt) levels in PBMCs DNA from cancer patients were previously observed [8, 12, 13], where low levels of global DNA methylation was related to future cancer development and therefore might be considered as a potentially useful epigenetic marker for cancer risk [8]. Cancer is a major public health issue [14]. Among other types of cancer, primary liver cancers, i.e. hepatocellular carcinoma (HCC) and cholangiocarcinoma (CC), and colon cancer, are common malignancies worldwide [14, 15]. In particular, the worldwide mortality rate of liver cancer is 14.6% in males and 5.7% in females, and the mortality of colon cancer is 9.7% in males and 7.0% in females [14].

**Fig 1:**
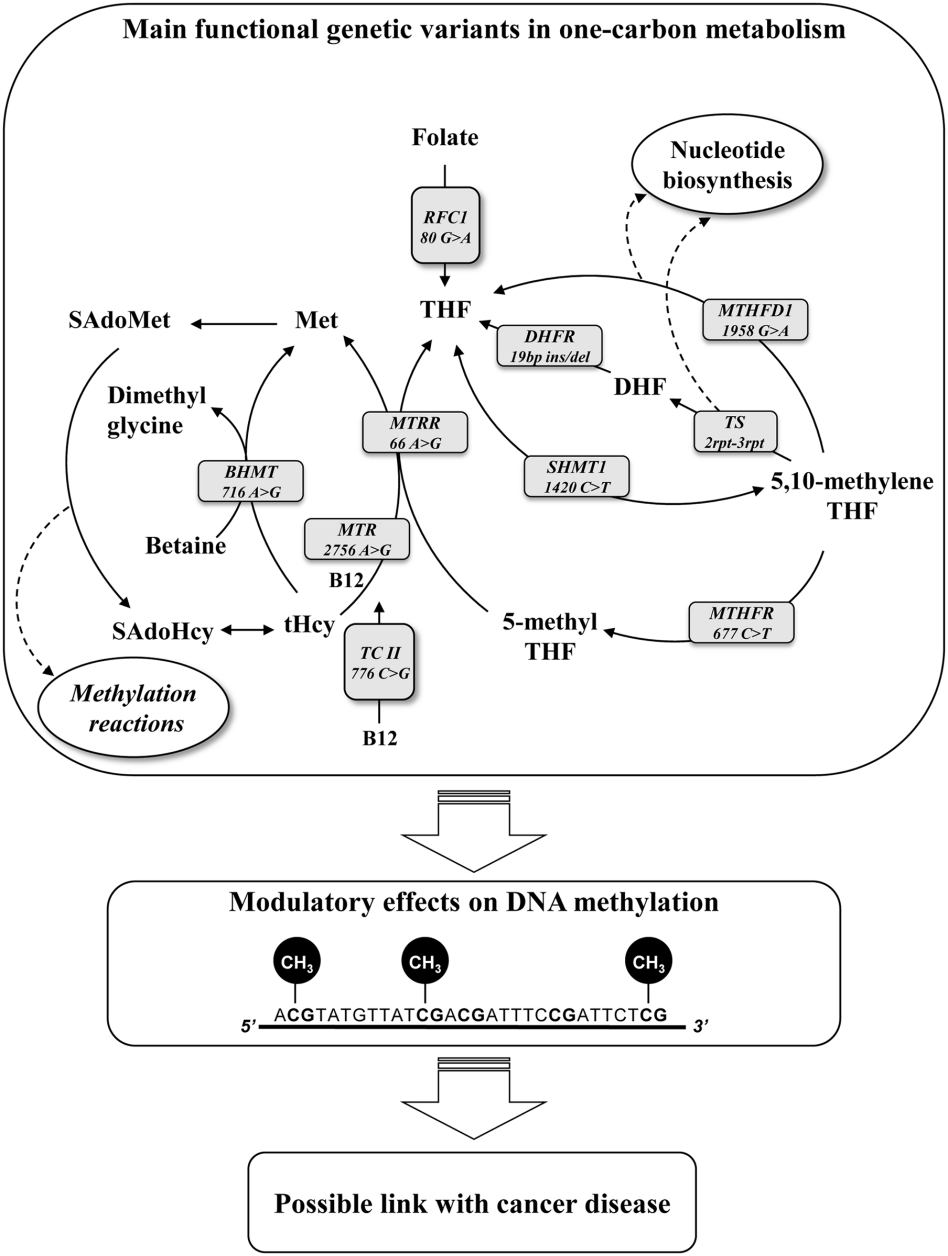
Hypothesis for a role of main functional genetic variants in one-carbon metabolism and cancer risk through DNA methylation. Polymorphic genetic variants in one-carbon enzymes can affect the balance between biological methylation and nucleic acids synthesis pathways inducing an aberrant DNA methylation and eventually leading to cancer development. BHMT, betaine-homocysteine S-methyltransferase; DHFR, dihydrofolate reductase; MTHFD1, methylenetetrahydrofolate dehydrogenase 1; MTHFR, methylenetetrahydrofolate reductase; MTR, 5-methyltetrahydrofolate-homocysteine methyltransferase; MTRR, 5-methyltetrahydrofolate-homocysteine methyltransferase reductase; RFC1, reduced folate carrier 1; SHMT1, serine hydroxymethyltransferase 1; TCII, transcobalamin II; THF, tetrahydrofolate, and TS, thymidylate synthase.

A potential underlying mechanism in colon and liver carcinogenesis relates to one-carbon metabolism for its function in providing methyl groups for DNA methylation [2]. A research hypothesis is that functional genetic defects in one-carbon enzymes, by inducing an aberrant DNA methylation, may eventually lead to cancer development, though evidences in this regard are still limited (Fig 1) [16].

Aim of the present study was precisely to evaluate the possible association among the most common, functional genetic variants of one carbon-metabolism enzymes, global DNA methylation and their potential association with risk for primary liver and colon cancer.

## Materials and methods

### Study subjects

The study was approved by the Institutional Review Board Ethical Committee of the University of Verona School of Medicine Hospital (Verona, Italy). Written informed consent was obtained from each patient after a detailed explanation of the study.

Four hundred and sixty-five subjects were enrolled, precisely 102 cancer patients (38 HCC, 21 CC, and 43 colon cancer) and 363 cancer-free subjects [17]. The patients affected by cancer were enrolled from April 2009 to March 2013 among those referring to the Division of General and Hepatobiliary Surgery of the Verona University Hospital, for curative surgery intervention. The patients admitted to the study were ≥18 years old with the following surgical resectability criteria: preserved liver function, class A Child-Pugh score, absence of extrahepatic metastases. Exclusion criteria comprised: a coexisting human immunodeficiency (HIV), hepatitis B (HBV) or hepatitis C (HCV) viruses infection; presence of relevant concurrent medical conditions such as acute or chronic inflammatory diseases or haematological disorders, autoimmune liver diseases and hemochromatosis, decompensate liver cirrhosis (Child-Pugh B, C). A trained physician recorded a detailed clinical history data reporting also lifestyle habits. The cancer-free control group was of 363 age- and sex-matched subjects [17]. Key eligibility criteria were age ≥18 years, absence of neoplasia of any type, no history of viral infections (HBV, HCV) and absence of other relevant medical conditions. All subjects under B vitamins supplementation and/or using drugs known to interfere with folate-related one-carbon metabolism in the month before enrolment were excluded.

### Biochemical analysis

Samples of venous blood were drawn from each subject after an overnight fasting for a complete blood count and determination of serum C-reactive protein (CRP), creatinine, aspartate transaminase (AST), alanine transaminase (ALT), gamma-glutamyltranspeptidase (gGT), alkaline phosphatase (ALP), total bilirubin, albumin, glicemia, total cholesterol, triglycerides, prothrombin time-international ratio (PT-INR), ferritin, and tests for hepatitis B and C viruses serology, by using routine laboratory test analyses. Plasma folate and vitamin B12 were measured by an automated chemiluminescence method (ChironDiagnostics, East Walpole, MA) and total plasma homocysteine (tHcy) concentrations were determined by high-performance liquid chromatography (HPLC) with fluorescent detection [18].

### Genotyping of one-carbon metabolism genes

From each subject venous blood was drawn into Vacutainer^®^ tubes containing EDTA as anticoagulant after an overnight fasting and DNA was extracted by Wizard Genomic DNA Purification Kit (Promega Corporation, Fitchburg, WI, USA). The one-carbon metabolism gene variants most commonly described with a functional effect were analyzed by different methods, as follows: *DHFR* 19bp ins/del (rs70991108)[19] and *TS* 2rpt-3rpt by PCR[20], *MTHFD1* 1958 G>A (rs2236225)[21], *MTHFR* 677 C>T (rs1801133)[22], *MTR* 2756A>G (rs12749581)[23], *MTRR* 66 A>G (rs1801394)[24], *RFC1* 80G>A (rs1051266)[25], and *SHMT1* 1420 C>T (rs1979277)[26] by PCR-restriction fragment length polymorphism assays, and the *BHMT* 716 A>G (rs3733890) and *TCII* 776 C>G (rs1801198) by allelic discrimination Real Time-PCR technology by using the C_11646606–20 assay and the C_325467–10 Taqman^®^ SNP Genotyping assays, respectively (Applied Biosystems^™^, ABI Prism 7500). The mean genotyping call rate among all the studied gene variants was 97.2%.

### Global DNA methylation

Global DNA methylation was determined using a liquid chromatography/mass spectrometry (LC/MS/MS) method and mCyt levels expressed as percent (%)mCyt = [(mCyt)/(mCyt+Cyt)] x 100, as previously described [7, 27, 28]. The analysis was performed in the PBMC DNA of all the cancer-free subjects (n = 363) and in a subgroup of cancer patients (n = 62). Briefly, global DNA was extracted and hydrolyzed to nucleosides using 2 units of nuclease P1, 0.002 units of venom phosphodiesterase I and 0.5 units of alkaline phosphatase. Isotope-labeled internal standards for deoxycytidine and 5-methyl-deoxycytidine were added to samples before running in a 3200 Q Trap MS-MS system coupled with an Agilent 1100 Series liquid chromatograph.

### Statistical analysis

All the statistical computations were performed by using the IBM SPSS Statistics software version 22 for Windows (IBM Inc., Armonk, NY, USA). Continuous variables were expressed as mean values and standard deviations (SD), while those showing a skewed distribution were log-transformed and, thus, expressed as geometric means with 95% confidence interval (CI). Continuous variables were tested by Student’s *t*-test or analysis of variance (ANOVA) with Tukey's post-hoc comparison when appropriate. Categorical variables were analyzed using a χ^2^-test. All genotype distributions were assessed according to Hardy-Weinberg equilibrium. The strength of association of gene variants with the risk of cancer was estimated by means of sex- and age-adjusted logistic regression and Odds Ratios (ORs) with 95% CI are reported. A value of p<0.05 was considered significant.

## Results

### Clinical characteristics of the subjects

Table 1 reports the main clinical and biochemical characteristics of the cancer patients and cancer-free subjects and, as shown, the two groups were age- and sex-matched. No differences were observed in terms of plasma folate and vitamin B12 concentrations between cases and controls while the groups differed for CRP, haemoglobin, platelets count, AST, ALT, ALP, gGT, homocysteine, albumin, total cholesterol, triglycerides, creatinine, and ferritin levels (Table 1). Serologic tests for HBV and HCV were negative for all patients according to the enrolment criteria, and indexes of hepatic function confirmed a compensated status of liver disease in all patients (Table 1).

**Table 1:** Clinical and biochemical characteristics of cancer patients and cancer-free subjects.

	Reference values	Cancer patients	Cancer-free subjects	p-value°
		n = 102	n = 363	
**Age (years)**		66.2 (10.9)	65.0 (5.9)	0.139
**Gender (% males)**		66.7%	70.5%	0.455^§^
**CRP (mg/L)**	<5.0	7.79 (5.75–10.18)	2.89 (2.61–3.13)	< 0.0001
**Hb (g/dL)**	13.5–16.0	13.1 (1.72)	14.2 (1.27)	< 0.0001
**MCV (fL)**	86.0–98.0	91.2 (6.68)	91.2 (5.36)	0.969
**WBC (10**^**9**^**/L)**	4.3–10.0	7.00 (2.77)	6.89 (1.70)	0.701
**Platelet count (10**^**9**^**/L)**	150–400	254 (111)	212 (61)	< 0.0001
AST (U/L)*	8–50	40.9 (35.2–47.5)	21.3 (20.5–22.2)	< 0.0001
ALT (U/L)*	8–45	36.2 (30.0–43.4)	23.3 (22.2–24.5)	< 0.0001
ALP (U/L)*	30–130	98.5 (87.4–111.1)	74.4 (72.2–76.7)	< 0.0001
gGT (U/L)*	<50	73.7 (60.3–89.1)	30.9 (28.8_33.1)	< 0.0001
Total bilirubin (mg/dL)*	0.11–1.05	0.66 (0.59–0.74)	0.67 (0.64–0.70)	0.852
PT (INR)*	0.82–1.14	2.25 (1.05–1.12)	2.39 (1.06–1.12)	0.773
**Albumin (g/L)**	35–50	39.8 (6.00)	41.8 (3.59)	< 0.0001
**Total cholesterol (mg/dL)**	<200	173.4 (48.7)	231.3 (44.0)	< 0.0001
Triglycerides (mg/dL)*	<150	116.8 (107.8–126.5)	142.6 (135.6–148.4)	< 0.0001
Creatinine (mg/dL)*	0.59–1.29	0.85 (0.79–0.92)	1.09 (1.06–1.13)	< 0.0001
**Glucose (mmol/L)**	3.5–5.5	5.85 (1.41)	6.17 (1.94)	0.121
Folate (nmol/L)*	10.4–42.4	13.2 (10.9–15.8)	12.1 (11.6–12.7)	0.390
tHcy (μg/L)*	< 15.0	11.9 (10.2–13.9)	15.8 (15.2–16.4)	<0.0001
VitaminB12 (pmol/L)*	142–724	323.8 (290.0–361.4)	287.2 (275.9–301.9)	0.056
Ferritin (μg/L)*	30–400	98.5 (89.1–108.9)	164.0 (127.7–210.6)	< 0.0001

Continuous variables with normal distribution are expressed as mean (standard deviation).

*: log-transformed variables are shown as geometric mean with 95% confidence interval

°: by Student’s t-test for comparison between cancer patients and cancer-free patients

^§^: χ-squared test

### One-carbon genetic variants and cancer risk

**Fig 2:**
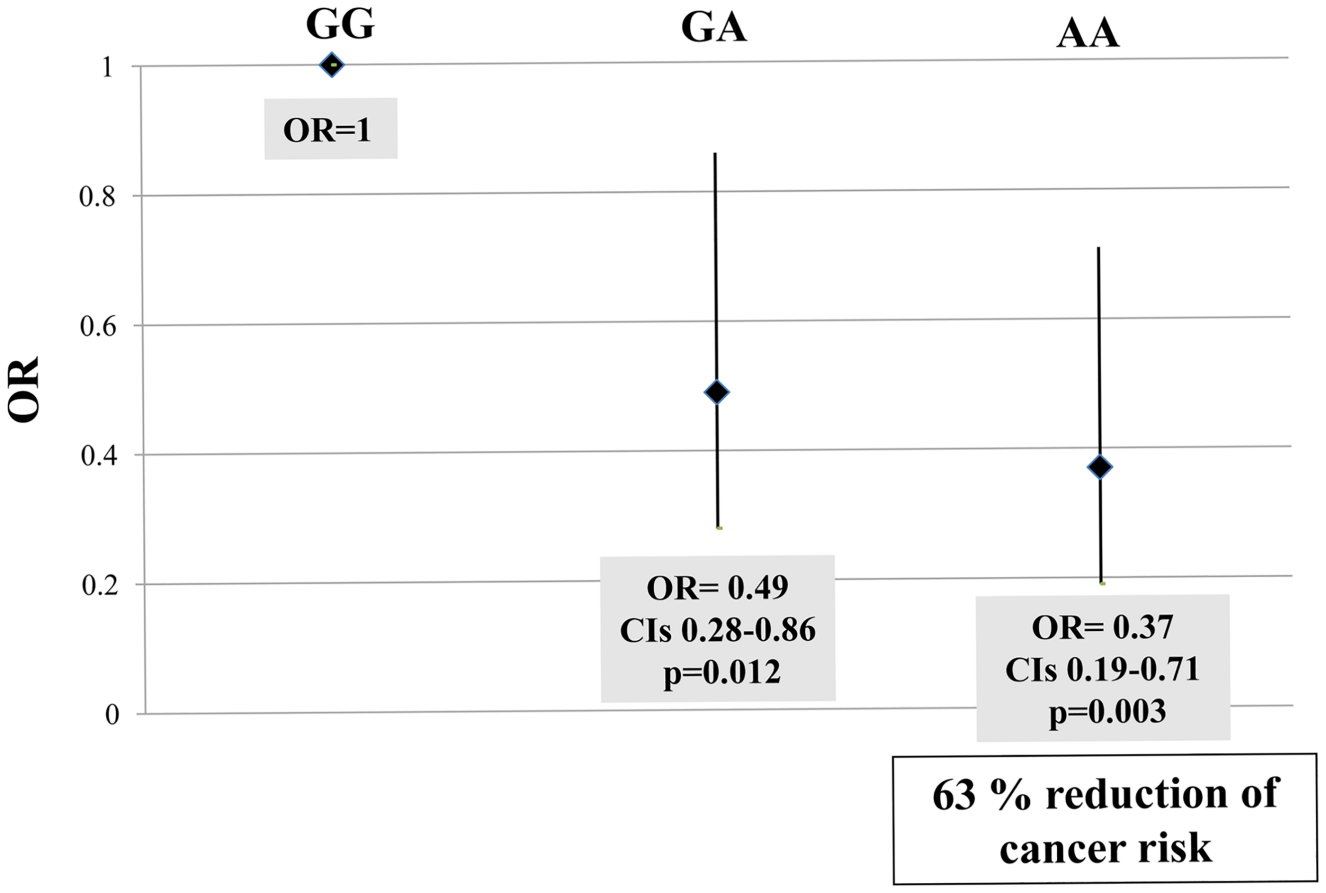
*MTHFD1* 1958G>A genotypes and cancer risk. The *MTHFD1* 1958A allele is associated to a lower cancer risk as expressed by means of Odds Ratio (OR) adjusted for sex and age.

The genotype distribution of the gene polymorphic variants was in agreement with the Hardy-Weinberg equilibrium both among cases and controls, except for the distribution, in the cancer group, of the *MTRR* 66A>G that was, therefore, excluded from subsequent analysis. Table 2 reports the distribution of polymorphic variants frequencies in cancer patients and in cancer-free subjects. As shown, there were no differences in the genotype distribution for all of the one-carbon pathway polymorphic variants analysed except for the *MTHFD1*1958AA genotype that was significantly less frequent among cancer patients as compared to cancer-free subjects (p = 0.008) and such difference was confirmed even after adjustments for sex and age (p = 0.007). The association with risk of cancer was then evaluated by setting the *MTHFD1* 1958GG genotype subjects as the reference group (OR = 1). The presence of the 1958A allele was associated with a lower risk for cancer in a graded manner so that the heterozygous variant, i.e. the 1958GA, was associated with a 51% risk reduction (OR = 0.49, CI 0.28–0.86, p = 0.012) and the 1958AA homozygous genotype was associated to a 63% reduction of cancer risk (OR = 0.37, CI 0.19–0.71, p = 0.003) (Fig 2). The risk for cancer was then analysed taking into account the 1958A allele carriership and the presence of the 1958A allele was associated to a lower cancer risk (OR = 0.46, CI 0.28–0.78, p = 0.005).

**Table 2:** Comparison of polymorphic variants frequencies between cancer patients and cancer-free subjects.

	Cancer patients N = 102			Cancer-free subjects N = 363			p-value
	w/w	w/m	m/m	w/w	w/m	m/m	
***BHMT* 716 G>A**	46.4	44.3	9.3	44.8	40.3	14.8	0.355
***DHFR* 19bp ins/del**	41.2	42.3	16.5	35.3	49.4	15.3	0.180
***MTHFD1* 1958 G>A**	29.6	49.0	21.4	16.2	52.9	30.9	0.007 *
***MTHFR* 677 C>T**	41.2	39.2	19.6	34.5	49.6	15.9	0.189
***MTR* 2756 A>G**	67.3	31.6	1.0	66.7	28.6	4.8	0.226
***RFC1* 80 G>A**	25.5	56.1	18.4	25.1	51.0	23.9	0.486
***SHMT1* 1420 C>T**	44.2	46.3	9.5	53.9	40.7	5.3	0.136
***TCII* 776 C>G**	32.7	50.0	17.3	31.5	51.4	17.1	0.967
***TS* 2rpt/3rpt** **	19.6	45.4	35.1	20.2	47.2	31.7	0.887

Abbreviations: w = major allele, m = minor allele, N.S. = not statistically significant;

* after adjustment for age and sex;

** 0.9% of cancer-free subjects were carriers of the 2rpt/4rpt and 3rpt/4rpt genotype.

The analysis for the distribution between cancer patients and cancer-free subjects did not detect any differences for the other polymorphic variants analysed.

### The *MTHFD1* 1958G>A genotypes according to cancer type and cancer risk

The *MTHFD1* 1958G>A genotype frequencies were then analysed according to the different types of cancer. Among HCC patients the frequencies were 24.3% (GG), 43.2% (GA) and 32.4% (AA); among CC patients the frequencies were 31.6% (GG), 57.9% (GA) and 10.5% (AA); as for the colon cancer patients group, the frequencies were 31.7% (GG), 52.2% (GA) and 17.1% (AA).

**Fig 3:**
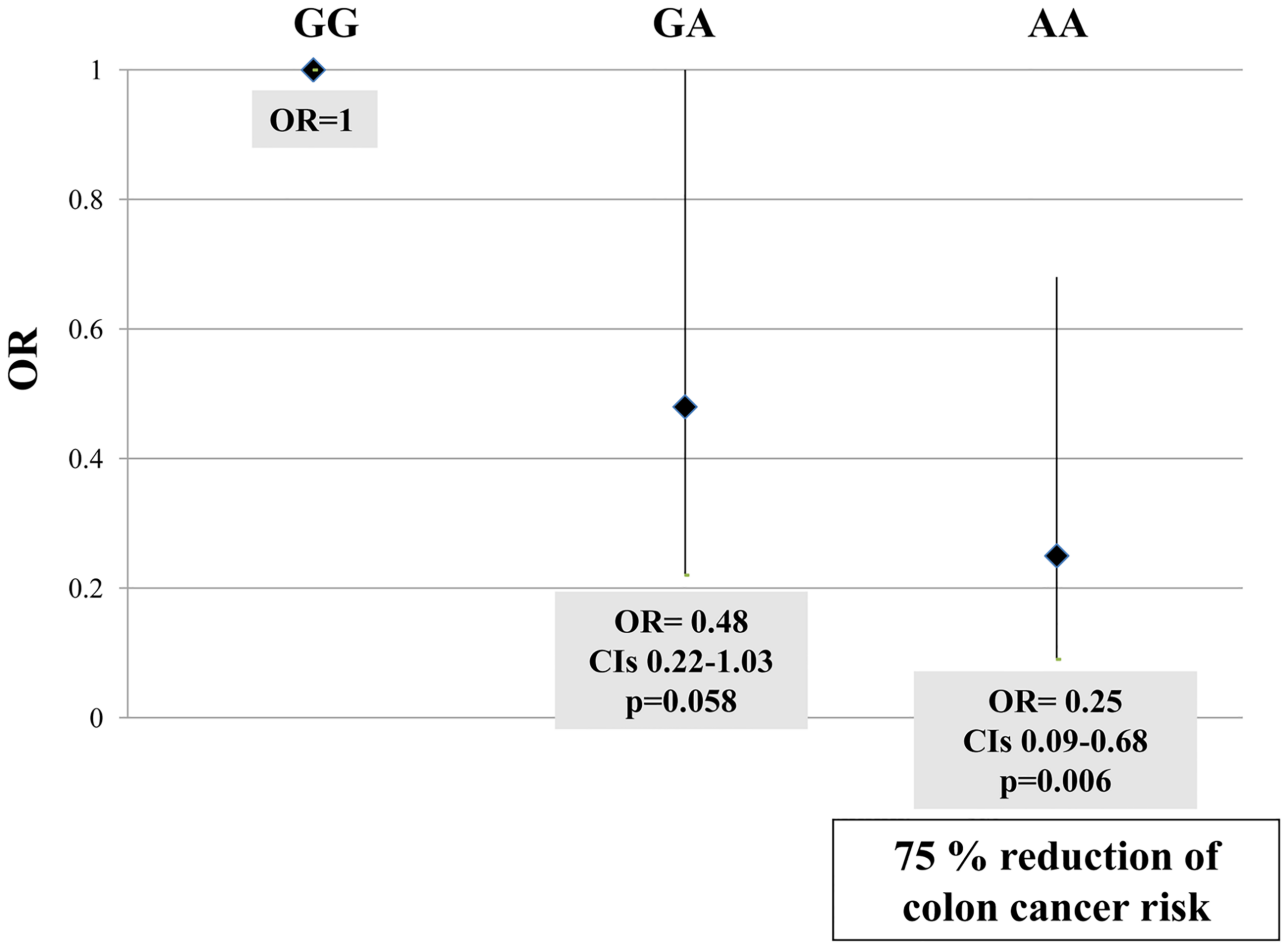
*MTHFD1* 1958G>A genotypes and colon cancer risk. The *MTHFD1*1958AA genotype is associated to a significantly reduced colon cancer risk by means of Odds Ratio (OR) adjusted for sex and age.

The *MTHFD1* 1958AA homozygous genotype was significantly less frequent among patients affected by colon cancer as compared to cancer-free subjects (17.1% *versus* 30.9%, p = 0.025) and this difference was confirmed when considering *MTHFD1* 1958A allele carriership (68.3% in colon cancer *versus* 83.8% in controls, p = 0.014). In HCC and CC patients no statistically significant differences were observed in terms of distribution, as compared to cancer-free subjects. The association with risk of colon cancer was evaluated by setting the *MTHFD1* 1958GG subjects as the reference group (OR = 1) and the adjustment for sex and age was then performed. When the heterozygous *MTHFD1* 1958GA variant was evaluated according to cancer risk, the analysis did not reach statistical significance (OR = 0.48, CI 0.22–1.03, p = 0.058) whereas the homozygotic condition, i.e. 1958AA, was associated to a 75% reduction of colon cancer risk (OR = 0.25, CI 0.09–0.68, p = 0.006) (Fig 3). Moreover, the 1958A allele carriership was linked to a noticeable lower risk for colon cancer (OR = 0.45, CI 0.27–0.76, p = 0.003).

### Global methylation in PBMCs DNA

**Fig 4:**
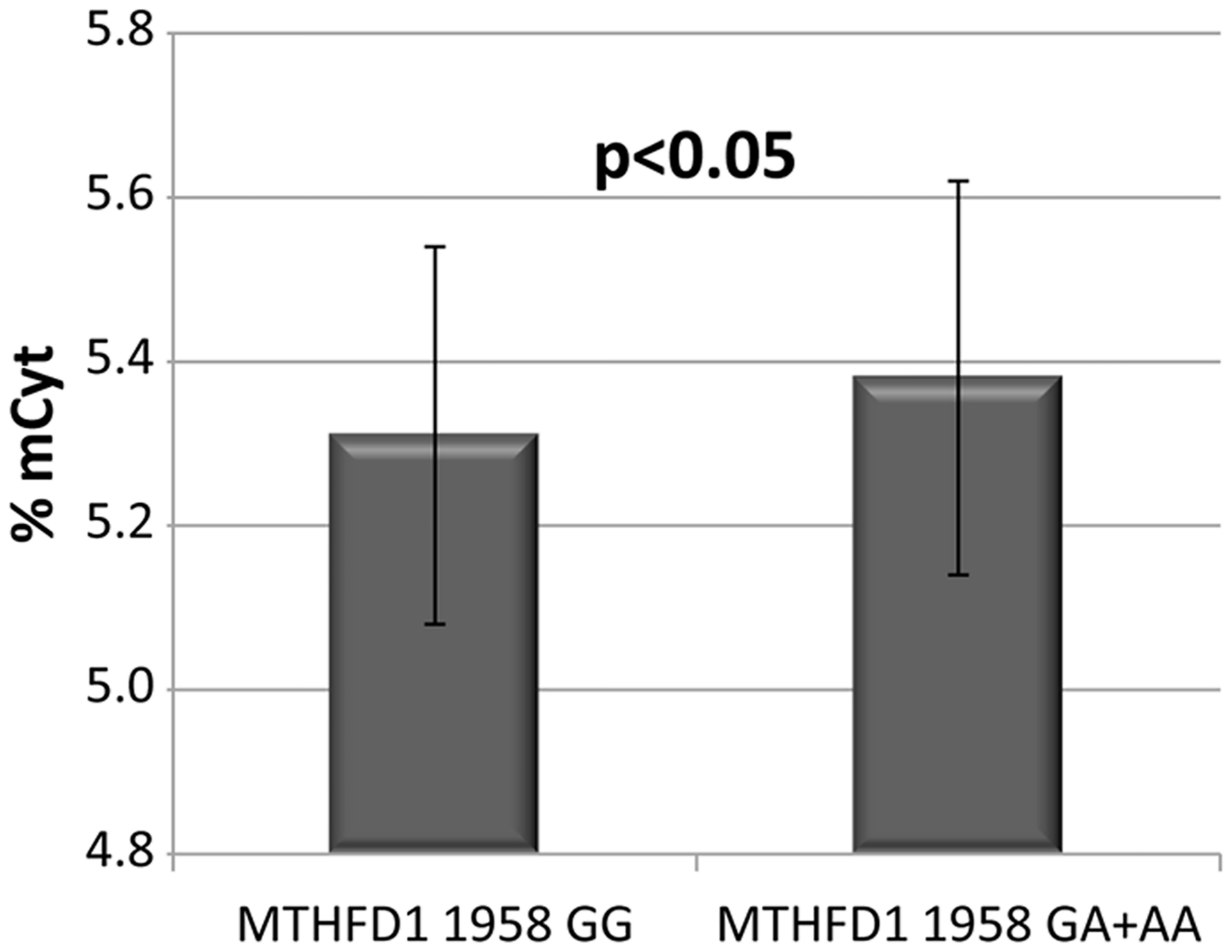
Global DNA methylation levels in PBMCs according to the *MTHFD1* 1958G>A genotypes. Global DNA methylation levels according to the *MTHFD1* 1958G>A genotypes in cancer patients and cancer-free subjects. GG: n = 72, GA+AA: n = 332. The error bar represents standard deviation (SD).

Methylcytosine was measured in PBMCs of a subgroup of subjects and no significant differences were observed between cancer- (n = 62) and cancer-free subjects (n = 363) (5.34% versus 5.38%, p = 0.38). A similar level of mCyt content characterized the three groups affected by the different types of cancer (HCC 5.34%, CC 5.33%, colon cancer 5.35%, p>0.2). The global DNA methylation in PBMCs was then analysed according to the *MTHFD1* 1958G>A genotypes. When the cancer patients group was considered as a whole, no differences were associated to mCyt levels among the three genotypes, as compared to the cancer-free subjects (p = 0.13). Then the mCyt levels were analyzed according to the presence of the 1958A allele (A carriers *versus* GG genotype) in all the study subjects (cancer patients and cancer-free subjects). The A allele carriers and the GG genotype subjects varied significantly for mCyt levels (5.38% *versus* 5.31%, p = 0.048) (Fig 4). Then mCyt levels were analysed according to *MTHFD1* 1958G>A genotypes in the three different type of cancer. Among colon cancer patients (n = 15) mCyt levels resulted higher in *MTHFD1* 1958A carriers as compared with GG genotype, although the difference did not reach the statistical significance (5.42% *versus* 5.10% respectively, p = 0.086).

## Discussion

Ten major polymorphisms related to one-carbon metabolism genes were analysed for the possible association with cancer risk, in particular HCC, CC and colon cancer risk. No differences in genotype distribution for all of the ten polymorphic variants analysed was, however, observed, neither there was an association with cancer risk, except for the *MTHFD1* 1958G>A. Furthermore, no differences were observed for folate and vitamin B12 levels between cancer and cancer-free subjects. This finding was, however, not surprising considering that both cancer and cancer-free subjects show folate and vitamin B12 status within a normality range.

This study demonstrates that the *MTHFD1* 1958GG genotype shows a higher frequency among cancer patients and it is associated, in all the study subjects, to PBMCs DNA hypomethylation as compared to the A allele carriers.

**Fig 5:**
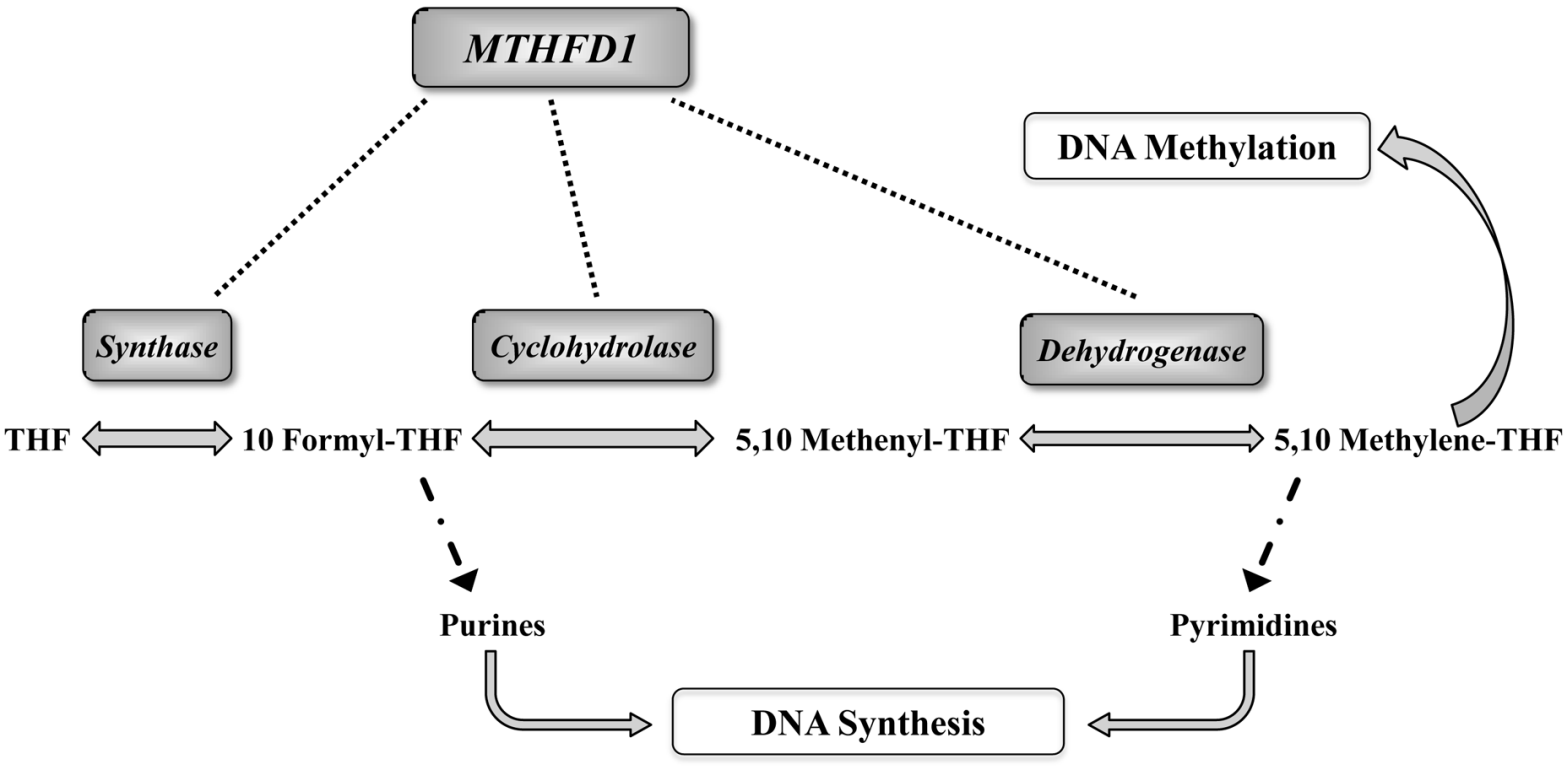
Pattern of reactions and functions of Methylene tetrahydrofolate dehydrogenase 1 (MTHFD1). The MTHFD1 enzyme catalyzes three sequential and reversible reactions in the pathway of conversion of tetrahydrofolate (THF), the active form of folate, into substrates essential for the *de novo* purine and thymidylate synthesis.

One-carbon metabolism is an essential crossroad in the balance between nucleotide synthesis and methylation reactions including that of DNA, known to be a key epigenetic mechanism in carcinogenesis [2, 12, 13]. Moreover, functional genetic variants of genes pertaining to one-carbon pathway are considered as potential risk factors for cancer disease (Fig 1) [5, 29]. Few data are available, so far concerning the mechanisms underlying the role of one-carbon metabolism variants in affecting cancer risk through the modulation of DNA methylation [8, 12, 13]. The MTHFD1 enzyme catalyzes three sequential and reversible reactions in the pathway of conversion of tetrahydrofolate (THF), the active form of folate, into substrates essential for the *de novo* purine and thymidylate synthesis [30] (Fig 5). Moreover, the MTHFD1 indirectly provides one-carbon units for methylation reactions by the synthesis of 5,10 methylene-THF[30]. Due to its role in nucleotide synthesis, the MTHFD1 enzyme may modulate cell division[31], it is therefore feasible that the functional alteration caused by the 1958G>A polymorphism may influence DNA synthesis reactions and cell development, eventually affecting carcinogenesis. The G>A substitution at position 1958 of the gene sequence leads to an arginine to glutamine substitution at 653 amino acid position in the enzyme synthase domain [21]. In a murine cell line model, the *MTHFD1* 1958AA genotype was associated to a 25% decreased purine synthesis [32] likely by affecting the supply of 10-formyl THF moieties for purine synthesis [33]. The function of the synthase domain was recently described to play an essential role in cellular proliferation by affecting nucleotide synthesis in rapidly dividing cells [34]. The *MTHFD1* 1958G>A variants have been mainly studied in relation to neural tube defects and embryonic development [31, 33, 35], but it has been also described as associated to cancer disease, although with not univocal results [3, 36, 37]. Wang and colleagues showed that the polymorphic 1958AA variant was linked to a significantly increased risk for gastric cancer as compared with the 1958GG or 1958AG genotypes [38], whereas most of the recent studies failed to demonstrate a significant association between the *MTHFD1*1958 G>A polymorphic variants and different types of cancers [3, 36, 37]. As it refers to colon cancer disease, several studies failed to find a statistically significant association between *MTHFD1* 1958 G>A and either colon cancer risk [5, 39, 40] or risk for colonic adenoma, a precursor lesion of colorectal cancer [41]. Interestingly, the present study showed a significantly different distribution of the *MTHFD1* 1958G>A genotypes between cancer and cancer-free subjects. In particular, there was a lower frequency of the *MTHFD1* 1958AA genotype among cancer patients as compared to cancer-free subjects and this difference was associated with a 63% reduction of the overall cancer risk. Even more intriguing was the significantly reduced *MTHFD1* 1958AA frequency in colon cancer patients as compared to controls and its association with a 75% reduction of colon cancer risk. Therefore our study demonstrates that the *MTHFD1* 1958AA is associated to a reduction for cancer risk, in particular for colon cancer. When global DNA methylation was analysed according to *MTHFD1* 1958G>A genotypes, the 1958GG genotype was either significantly more represented in patients affected by cancer, and associated with lower mCyt levels in PBMCs DNA. These results are in agreement with the previous observation of global DNA hypomethylation in cancer tissues [6, 12, 13] as well as in PBMCs DNA of patients affected by cancer [8]. In the subgroup of patients affected by colon cancer, the association between the presence of *MTHFD1* 1958GG genotype and DNA hypomethylation in PBMCs did not reach statistical significance probably due to the small sample size. The reported findings of the significantly lower frequency of the *MTHFD1* 1958AA genotype in patients affected by cancer disease and the association of this genotype with a lower risk for cancer appear suggestive for a protective role of this polymorphic variant, particularly in the development of colon cancer. The mechanism underlying this hypothesis may be a reduced synthase activity associated with the homozygous 1958AA genotype [32], which may slow down the cell proliferation processes and hinder the tumour initiation. Moreover, the present results suggest that both *MTHFD1* 1958AA and 1958GA genotypes could be protective for cancer through modulation of DNA methylation. In colon cancer patients the difference between mCyt levels in association with the 1958GG and the 1958 GA+AA genotypes respectively, may be due to the small sample size, thus further investigations are warranted.

In conclusion, the *MTHFD1* 1958AA genotype is less frequent among cancer patients and it is associated to a lower risk for cancer that is even more decreased specifically for colon cancer. Moreover, the association of A allele with the presence of higher DNA methylation levels in PBMCs suggests a possible protective role of this variant against DNA hypomethylation. On the other hand, the *MTHFD1* 1958GG genotype, more frequent among cancer patients, indicates a trend towards global PBMCs DNA hypomethylation as commonly observed in cancer. The link between the *MTHFD1* 1958AA genotype with a decreased cancer risk may be explained by its possible modulatory effect between DNA methylation and nucleotide synthesis. Further studies are certainly needed to better unravel the role of *MTHFD1* 1958 A>G in cancer risk.

## References

[1] Choi SW, Mason JB. Folate status: effects on pathways of colorectal carcinogenesis. The Journal of nutrition. 2002;132(8 Suppl):2413S–8S. . 12163703 10.1093/jn/132.8.2413S

[2] Friso S, Choi SW. Gene-nutrient interactions and DNA methylation. The Journal of nutrition. 2002;132(8 Suppl):2382S–7S. . 12163697 10.1093/jn/132.8.2382S

[3] Zee RY, Rose L, Chasman DI, Ridker PM. Genetic variation of fifteen folate metabolic pathway associated gene loci and the risk of incident head and neck carcinoma: the Women's Genome Health Study. Clinica chimica acta; international journal of clinical chemistry. 2013;418:33–6. doi: 10.1016/j.cca.2012.11.030 23276522 10.1016/j.cca.2012.11.030 PMC3582777

[4] Babyshkina N, Malinovskaya E, Nazarenko M, Koval M, Gervas P, Potapova O, et al The effect of folate-related SNPs on clinicopathological features, response to neoadjuvant treatment and survival in pre- and postmenopausal breast cancer patients. Gene. 2013;518(2):397–404. doi: 10.1016/j.gene.2012.12.095 . 23296054 10.1016/j.gene.2012.12.095

[5] Chen J, Kyte C, Valcin M, Chan W, Wetmur JG, Selhub J, et al Polymorphisms in the one-carbon metabolic pathway, plasma folate levels and colorectal cancer in a prospective study. International journal of cancer Journal international du cancer. 2004;110(4):617–20. doi: 10.1002/ijc.20148 . 15122597 10.1002/ijc.20148

[6] Esteller M. Epigenetics in cancer. The New England journal of medicine. 2008;358(11):1148–59. Epub 2008/03/14. doi: 10.1056/NEJMra072067 . 18337604 10.1056/NEJMra072067

[7] Friso S, Choi SW, Girelli D, Mason JB, Dolnikowski GG, Bagley PJ, et al A common mutation in the 5,10-methylenetetrahydrofolate reductase gene affects genomic DNA methylation through an interaction with folate status. Proceedings of the National Academy of Sciences of the United States of America. 2002;99(8):5606–11. Epub 2002/04/04. doi: 10.1073/pnas.062066299 11929966 10.1073/pnas.062066299 PMC122817

[8] Friso S, Udali S, Guarini P, Pellegrini C, Pattini P, Moruzzi S, et al Global DNA hypomethylation in peripheral blood mononuclear cells as a biomarker of cancer risk. Cancer epidemiology, biomarkers & prevention: a publication of the American Association for Cancer Research, cosponsored by the American Society of Preventive Oncology. 2013 Epub 2013/01/10. doi: 10.1158/1055-9965.EPI-12-0859 . 23300023 10.1158/1055-9965.EPI-12-0859 PMC3596466

[9] Feinberg AP. Phenotypic plasticity and the epigenetics of human disease. Nature. 2007;447(7143):433–40. Epub 2007/05/25. doi: 10.1038/nature05919 . 17522677 10.1038/nature05919

[10] Ehrlich M. Cancer-linked DNA hypomethylation and its relationship to hypermethylation. Curr Top Microbiol Immunol. 2006;310:251–74. Epub 2006/08/17. . 16909914 10.1007/3-540-31181-5_12

[11] Woo HD, Kim J. Global DNA hypomethylation in peripheral blood leukocytes as a biomarker for cancer risk: a meta-analysis. PloS one. 2012;7(4):e34615 doi: 10.1371/journal.pone.0034615 22509334 10.1371/journal.pone.0034615 PMC3324531

[12] Udali S, Guarini P, Moruzzi S, Ruzzenente A, Tammen SA, Guglielmi A, et al Global DNA methylation and hydroxymethylation differ in hepatocellular carcinoma and cholangiocarcinoma and relate to survival rate. Hepatology. 2015;62(2):496–504. doi: 10.1002/hep.27823 . 25833413 10.1002/hep.27823

[13] Moruzzi S, Udali S, Ruzzenente A, Guglielmi A, Guarini P, Martinelli N, et al The RFC1 80G>A, among Common One-Carbon Polymorphisms, Relates to Survival Rate According to DNA Global Methylation in Primary Liver Cancers. PloS one. 2016;11(12):e0167534 doi: 10.1371/journal.pone.0167534 27936032 10.1371/journal.pone.0167534 PMC5147923

[14] Ferlay J, Shin HR, Bray F, Forman D, Mathers C, Parkin DM. Estimates of worldwide burden of cancer in 2008: GLOBOCAN 2008. International journal of cancer Journal international du cancer. 2010;127(12):2893–917. doi: 10.1002/ijc.25516 . 21351269 10.1002/ijc.25516

[15] O'Connell JB, Maggard MA, Ko CY. Colon cancer survival rates with the new American Joint Committee on Cancer sixth edition staging. Journal of the National Cancer Institute. 2004;96(19):1420–5. doi: 10.1093/jnci/djh275 . 15467030 10.1093/jnci/djh275

[16] Choi SW, Mason JB. Folate and carcinogenesis: an integrated scheme. The Journal of nutrition. 2000;130(2):129–32. . 10720158 10.1093/jn/130.2.129

[17] Girelli D, Friso S, Trabetti E, Olivieri O, Russo C, Pessotto R, et al Methylenetetrahydrofolate reductase C677T mutation, plasma homocysteine, and folate in subjects from northern Italy with or without angiographically documented severe coronary atherosclerotic disease: evidence for an important genetic-environmental interaction. Blood. 1998;91(11):4158–63. . 9596662

[18] Araki A, Sako Y. Determination of free and total homocysteine in human plasma by high-performance liquid chromatography with fluorescence detection. Journal of Chromatography. 1987;422:43–52. 3437026 10.1016/0378-4347(87)80438-3

[19] Johnson WG, Stenroos ES, Spychala JR, Chatkupt S, Ming SX, Buyske S. New 19 bp deletion polymorphism in intron-1 of dihydrofolate reductase (DHFR): a risk factor for spina bifida acting in mothers during pregnancy? American journal of medical genetics Part A. 2004;124A(4):339–45. doi: 10.1002/ajmg.a.20505 . 14735580 10.1002/ajmg.a.20505

[20] Ulrich CM, Bigler J, Bostick R, Fosdick L, Potter JD. Thymidylate synthase promoter polymorphism, interaction with folate intake, and risk of colorectal adenomas. Cancer research. 2002;62(12):3361–4. . 12067974

[21] Hol FA, van der Put NM, Geurds MP, Heil SG, Trijbels FJ, Hamel BC, et al Molecular genetic analysis of the gene encoding the trifunctional enzyme MTHFD (methylenetetrahydrofolate-dehydrogenase, methenyltetrahydrofolate-cyclohydrolase, formyltetrahydrofolate synthetase) in patients with neural tube defects. Clinical genetics. 1998;53(2):119–25. . 9611072 10.1111/j.1399-0004.1998.tb02658.x

[22] Frosst P, Blom HJ, Milos R, Goyette P, Sheppard CA, Matthews RG, et al A candidate genetic risk factor for vascular disease: a common mutation in methylenetetrahydrofolate reductase. Nature genetics. 1995;10(1):111–3. doi: 10.1038/ng0595-111 . 7647779 10.1038/ng0595-111

[23] Klerk M, Lievers KJ, Kluijtmans LA, Blom HJ, den Heijer M, Schouten EG, et al The 2756A>G variant in the gene encoding methionine synthase: its relation with plasma homocysteine levels and risk of coronary heart disease in a Dutch case-control study. Thrombosis research. 2003;110(2–3):87–91. . 12893022 10.1016/s0049-3848(03)00341-4

[24] Jacques PF, Bostom AG, Selhub J, Rich S, Ellison RC, Eckfeldt JH, et al Effects of polymorphisms of methionine synthase and methionine synthase reductase on total plasma homocysteine in the NHLBI Family Heart Study. Atherosclerosis. 2003;166(1):49–55. . 12482550 10.1016/s0021-9150(02)00204-6

[25] Chango A, Emery-Fillon N, de Courcy GP, Lambert D, Pfister M, Rosenblatt DS, et al A polymorphism (80G->A) in the reduced folate carrier gene and its associations with folate status and homocysteinemia. Molecular genetics and metabolism. 2000;70(4):310–5. doi: 10.1006/mgme.2000.3034 . 10993718 10.1006/mgme.2000.3034

[26] Heil SG, Van der Put NM, Waas ET, den Heijer M, Trijbels FJ, Blom HJ. Is mutated serine hydroxymethyltransferase (SHMT) involved in the etiology of neural tube defects? Molecular genetics and metabolism. 2001;73(2):164–72. doi: 10.1006/mgme.2001.3175 . 11386852 10.1006/mgme.2001.3175

[27] Friso S, Choi SW, Dolnikowski GG, Selhub J. A method to assess genomic DNA methylation using high-performance liquid chromatography/electrospray ionization mass spectrometry. Anal Chem. 2002;74(17):4526–31. Epub 2002/09/19. . 12236365 10.1021/ac020050h

[28] Tammen SA, Dolnikowski GG, Ausman LM, Liu Z, Sauer J, Friso S, et al Aging and alcohol interact to alter hepatic DNA hydroxymethylation. Alcoholism, clinical and experimental research. 2014;38(8):2178–85. doi: 10.1111/acer.12477 25070523 10.1111/acer.12477 PMC4146686

[29] Chang SC, Chang PY, Butler B, Goldstein BY, Mu L, Cai L, et al Single nucleotide polymorphisms of one-carbon metabolism and cancers of the esophagus, stomach, and liver in a chinese population. PLoS One. 2014;9(10):e109235 doi: 10.1371/journal.pone.0109235 25337902 10.1371/journal.pone.0109235 PMC4206280

[30] Hum DW, Bell AW, Rozen R, MacKenzie RE. Primary structure of a human trifunctional enzyme. Isolation of a cDNA encoding methylenetetrahydrofolate dehydrogenase-methenyltetrahydrofolate cyclohydrolase-formyltetrahydrofolate synthetase. The Journal of biological chemistry. 1988;263(31):15946–50. . 3053686

[31] Brody LC, Conley M, Cox C, Kirke PN, McKeever MP, Mills JL, et al A polymorphism, R653Q, in the trifunctional enzyme methylenetetrahydrofolate dehydrogenase/methenyltetrahydrofolate cyclohydrolase/formyltetrahydrofolate synthetase is a maternal genetic risk factor for neural tube defects: report of the Birth Defects Research Group. American journal of human genetics. 2002;71(5):1207–15. doi: 10.1086/344213 12384833 10.1086/344213 PMC385099

[32] Christensen KE, Rohlicek CV, Andelfinger GU, Michaud J, Bigras JL, Richter A, et al The MTHFD1 p.Arg653Gln variant alters enzyme function and increases risk for congenital heart defects. Human mutation. 2009;30(2):212–20. doi: 10.1002/humu.20830 . 18767138 10.1002/humu.20830

[33] Parle-McDermott A, Kirke PN, Mills JL, Molloy AM, Cox C, O'Leary VB, et al Confirmation of the R653Q polymorphism of the trifunctional C1-synthase enzyme as a maternal risk for neural tube defects in the Irish population. European journal of human genetics: EJHG. 2006;14(6):768–72. doi: 10.1038/sj.ejhg.5201603 . 16552426 10.1038/sj.ejhg.5201603

[34] Christensen KE, Deng L, Leung KY, Arning E, Bottiglieri T, Malysheva OV, et al A novel mouse model for genetic variation in 10-formyltetrahydrofolate synthetase exhibits disturbed purine synthesis with impacts on pregnancy and embryonic development. Human molecular genetics. 2013;22(18):3705–19. doi: 10.1093/hmg/ddt223 . 23704330 10.1093/hmg/ddt223

[35] Jiang J, Zhang Y, Wei L, Sun Z, Liu Z. Association between MTHFD1 G1958A polymorphism and neural tube defects susceptibility: a meta-analysis. PloS one. 2014;9(6):e101169 doi: 10.1371/journal.pone.0101169 24977710 10.1371/journal.pone.0101169 PMC4076264

[36] Lautner-Csorba O, Gezsi A, Erdelyi DJ, Hullam G, Antal P, Semsei AF, et al Roles of genetic polymorphisms in the folate pathway in childhood acute lymphoblastic leukemia evaluated by Bayesian relevance and effect size analysis. PloS one. 2013;8(8):e69843 doi: 10.1371/journal.pone.0069843 23940529 10.1371/journal.pone.0069843 PMC3734218

[37] Galbiatti AL, da Silva LM, Ruiz-Cintra MT, Raposo LS, Maniglia JV, Pavarino EC, et al Association between 11 genetic polymorphisms in folate-metabolising genes and head and neck cancer risk. European journal of cancer. 2012;48(10):1525–31. doi: 10.1016/j.ejca.2011.09.025 . 22051736 10.1016/j.ejca.2011.09.025

[38] Wang L, Ke Q, Chen W, Wang J, Tan Y, Zhou Y, et al Polymorphisms of MTHFD, plasma homocysteine levels, and risk of gastric cancer in a high-risk Chinese population. Clinical cancer research: an official journal of the American Association for Cancer Research. 2007;13(8):2526–32. doi: 10.1158/1078-0432.CCR-06-2293 . 17438114 10.1158/1078-0432.CCR-06-2293

[39] Eussen SJ, Vollset SE, Igland J, Meyer K, Fredriksen A, Ueland PM, et al Plasma folate, related genetic variants, and colorectal cancer risk in EPIC. Cancer epidemiology, biomarkers & prevention: a publication of the American Association for Cancer Research, cosponsored by the American Society of Preventive Oncology. 2010;19(5):1328–40. doi: 10.1158/1055-9965.EPI-09-0841 20447924 10.1158/1055-9965.EPI-09-0841 PMC2880712

[40] Levine AJ, Figueiredo JC, Lee W, Conti DV, Kennedy K, Duggan DJ, et al A candidate gene study of folate-associated one carbon metabolism genes and colorectal cancer risk. Cancer epidemiology, biomarkers & prevention: a publication of the American Association for Cancer Research, cosponsored by the American Society of Preventive Oncology. 2010;19(7):1812–21. doi: 10.1158/1055-9965.EPI-10-0151 20615890 10.1158/1055-9965.EPI-10-0151 PMC2950115

[41] Han SS, Sue LY, Berndt SI, Selhub J, Burdette LA, Rosenberg PS, et al Associations between genes in the one-carbon metabolism pathway and advanced colorectal adenoma risk in individuals with low folate intake. Cancer epidemiology, biomarkers & prevention: a publication of the American Association for Cancer Research, cosponsored by the American Society of Preventive Oncology. 2012;21(3):417–27. doi: 10.1158/1055-9965.EPI-11-0782 . 22253295 10.1158/1055-9965.EPI-11-0782

